# Increase in Serum Interleukin-1 Receptor Antagonist (IL-1ra) Levels after Wheelchair Half Marathon Race in Male Athletes with Spinal Cord Injury

**DOI:** 10.3390/jcm12227098

**Published:** 2023-11-15

**Authors:** Masumi Nakahama-Matsushima, Yoshi-ichiro Kamijyo, Yasunori Umemoto, Takamasa Hashizaki, Yukihide Nishimura, Kazunari Furusawa, Yohei Furotani, Fumihiro Tajima, Ken Kouda

**Affiliations:** 1 Department of Rehabilitation Medicine, School of Medicine, Wakayama Medical University, Wakayama 641-8509, Japan; nakahama@wakayama-med.ac.jp (M.N.-M.);; 2 Department of Rehabilitation Medicine, School of Medicine, Dokkyo Medical University, Mibu 321-0293, Japan; 3 Department of Rehabilitation Medicine, School of Medicine, Iwate Medical University, Yahaba 028-3695, Japan; 4 Department of Rehabilitation Medicine, Kibikogen Rehabilitation Center for Employment Injuries, Okayama 716-1241, Japan

**Keywords:** exercise, myokine, interleukin-6, neutrophil, parasports

## Abstract

Exercise increases the serum level of interleukin-6 (IL-6), which in turn stimulates the production of various inflammatory cytokine antagonists, such as interleukin-1 receptor antagonist (IL-1ra). Individuals with cervical spinal cord injury (CSCI) are at high risk of inflammatory conditions. This study compared the effects of wheelchair half marathon on the immune system of male athletes with CSCI and those with thoracic/lumber spinal cord injury (SCI). Neutrophil count, IL-1ra, IL-6, and various endocrine parameters were measured before, immediately and 1 h after the race in five CSCI and six SCI who completed the wheelchair marathon race. The percentage of neutrophils was significantly higher in CSCI immediately and 1 h after the race, compared with the baseline, and significantly higher in SCI at 1 h after the race. IL-6 was significantly higher immediately and 1 h after the race in SCI, whereas no such changes were noted in IL-6 in CSCI. IL-1ra was significantly higher at 1 h after the race in both SCI and CSCI. The race was associated with an increase in IL-1ra in both CSCI and SCI. These findings suggest wheelchair half marathon race increases IL-1ra even under stable IL-6 status in male CSCI individuals, and that such post-race increase in IL-1ra is probably mediated through circulatory neutrophils.

## 1. Introduction

Persons with cervical spinal cord injury (CSCI) are at high risk of low-grade inflammation associated with dysfunction of the sympathetic nervous system. Individuals with CSCI as well as persons with thoracic or lumber spinal cord injury (SCI) are encouraged to exercise regularly to improve general health status [1,2,3]. Evidence suggests that physical exercise has anti-inflammatory effects and thus tends to protect against chronic clinical disorders associated with low-grade systemic inflammation [4]. However, details of the anti-inflammatory system involved in such process remain elusive.

Regular exercise offers protection against all-cause mortality, primarily by protecting against the development/complications of cardiovascular diseases and type 2 diabetes mellitus. The pathophysiology of the latter disorders is thought to be associated with chronic low-grade systemic inflammation reflected by two- to three-fold increase in the serum levels of various cytokines [4]. Physical activity induces an increase in the systemic levels of various anti-inflammatory cytokines. Furthermore, the inflammatory response to strenuous exercise is well-documented and the coordinated action of cytokines is considered important in the regulation of inflammatory reactions involved in immune defense and muscle repair [5].

Early in this century, skeletal muscles were found to behave like an endocrine organ and secrete various cytokines during physical exercise, such as interleukin (IL)-6 [6]. Since then, exercise and skeletal muscle contractile activity has been linked to various physiological processes involved in maintaining health in humans [7]. In this regard, several research groups investigated the link between muscle contraction and humoral changes in the form of an “exercise factor”, and metabolic changes in other organs. For example, Pedersen et al. [8] suggested that cytokines and other peptides are produced, expressed, and released by muscle fibers and exert either paracrine or endocrine effects. Furthermore, the discovery of interleukin (IL)-6 release from contracting skeletal muscles added support to the effect of IL-6 on metabolism [8]. Muscle-derived IL-6 fulfills the criteria of an exercise factor and these endocrine factors were considered to serve important metabolic roles and collectively termed myokines [9]. Anti-inflammatory cytokines secreted by exercising muscles are also thought to contribute towards protection against cardiovascular disease and type 2 diabetes mellitus [6].

Exercise-induced increase in IL-6 stimulates the production of various inflammatory cytokine antagonists, such as IL-1ra [10,11]. To date, three members of the IL-1 gene family have been characterized: IL-1α, IL-1β, and IL-1 receptor antagonist (IL-1Ra). IL-lα and IL-1β are agonists and IL-1Ra is a specific receptor antagonist. IL-1α and IL-1β are typical proinflammatory cytokines, while IL-1ra acts as an antagonist of the IL-1 receptor type I (IL-1RI) and prevents IL-1 (both α and β)-dependent signaling. In the absence of IL-1ra, the activity of IL-1 is unopposed, promoting rampant inflammation [5]. Increased IL-1 production has been reported in patients with infections, intravascular coagulation, neuromuscular diseases, autoimmune disorders, trauma, ischemic diseases, UV radiation, and in healthy subjects after strenuous exercise [12]. The IL-1 family of ligands and receptors is primarily associated with acute and chronic inflammation [13]. Interleukin-1 (IL-1α and IL-1β) is the prototypic “multifunctional” cytokine and affects nearly every cell type, often in concert with other cytokines or small mediator molecules [12]. IL-1β is a systemic hormone-like mediator released from cells, whereas IL-lα is primarily a regulator of intracellular events and mediator of local inflammation. IL-1RI is an 80-kD glycoprotein found in endothelial cells, epithelial cells, smooth muscle cells, keratinocytes, epidermal dendritic cells, hepatocytes, fibroblasts, and T lymphocytes [12]. IL-1RI is heavily glycosylated and blockade of the glycosylation sites reduces the binding of IL-1. IL-1RI is regulated in inflammation and immune responses [12]. IL-1ra improves blood glucose levels, suppresses systemic inflammation, and prevents the progression to heart failure associated with acute hypertension, and has been proposed as one of the mechanisms for the beneficial effects of exercise on lifestyle-related diseases and heart failure [14,15].

Initial studies suggested that exercise involving upper limb muscles did not produce sufficient muscle activity to increase myokines [6,16,17]. However, in a series of studies from our laboratories, we demonstrated that 2 h arm crank ergometer exercise at 60% VO_2_max induced an increase in plasma IL-6 levels in individuals with spinal cord injury (SCI) [1] and that half- and full-marathons by wheelchair SCI athletes result in increases in IL-6 [18]. We also demonstrated an increase in plasma IL-6 levels after long and intensive exercise in patients with cervical spinal cord injury (CSCI) [19].

IL-1ra has various anti-inflammatory functions, similar to IL-6. IL-1ra is produced by various cell types, including neutrophils, macrophages, fibroblasts, and monocytes [20,21], and the production level [21] parallels that of IL-6 [11]. Previous studies have reported that exercise, such as marathon running and resistance training, increases serum IL-1ra levels [10,22]. It has also been reported that IL-1ra concentrations are lower in disease-prone groups, suggesting a deficit in the anti-inflammatory regulatory capacity in disease-prone athletes [23,24]. However, the exact mechanism of IL-1ra secretion during exercise is still poorly understood.

The level of circulating IL-6 increases exponentially up to 100-fold immediately after exercise, but falls later during the postexercise period [4,7,25,26,27]. Exercise provokes primarily an increase in IL-6, followed by an increase in IL-1ra and IL-10. Interestingly, the appearance of IL-6 in the circulation is by far the most marked and it precedes that of other cytokines [4].

Patients with CSCI have reduced muscle mass and a blunted plasma IL-6 increase following long and intensive exercise [19]. They are prone to developing various inflammatory conditions that often require treatment with high doses of anti-inflammatory agents [28,29]. Should wheelchair exercise increase the levels of anti-inflammatory substances, it will be important to encourage its use in such individuals with CSCI and low muscle mass.

Cortisol is known as the “stress hormone”. With regard to the cortisol-regulating system (hypothalamus, pituitary gland and adrenal glands, HPA axis), the hypothalamus first releases corticotropin-releasing hormone (CRH), which stimulates the pituitary gland to produce adrenocorticotropic hormone (ACTH). ACTH then stimulates the adrenal glands to produce and release cortisol. CRH modulates stress-induced autonomic, behavioral, and local inflammatory responses and the HPA axis stimulates cortisol excretion. In response to stress, the hypothalamus sends direct signals via the sympathetic nervous system to the adrenal glands, causing them to release adrenaline and noradrenaline. Injection of IL-6 increases plasma ACTH and plasma cortisol [11,30]. Moreover, both the pituitary corticotrophs and adrenocortical cells express IL-6 receptors, and IL-6 can induce an increase in cortisol, both directly and indirectly [11,30].

Moderate- to high-intensity exercise is known to increase circulating cortisol levels, although low-intensity exercise (40%) does not result in a significant increase in cortisol levels [31]. The effects of exercise on cortisol secretion in individuals with CSCI and SCI remain unknown and there is no information on the correlation between cortisol and IL-1ra in these individuals.

The present study was conducted to compare the effects of intensive exercise on IL-1ra, IL-6 and various hormones between individuals with SCI and those with CSCI. The main theme of the study was to determine whether wheelchair exercise enhances the immune systems of these two groups of athletes, with a particular emphasis on IL-1ra and related factors.

## 2. Methods

### 2.1. Participants

Five CSCI and six SCI athletes were invited to participate in the study following a detailed description of the study purpose and protocol and possible risks. All eleven accepted the invitation, and each signed the informed consent form and voluntarily participated in the study. All subjects completed the half marathon division of the 38th-Oita International Wheelchair Marathon Race in Japan. Table 1 provides details of the participants. There were no differences between the CSCI and SCI groups with respect to age and body mass index (BMI). The levels of spinal cord lesions were C5-8 and T3-L1 for CSCI and SCI, respectively. The selection criteria for the study were the following: (1) more than 1 year after spinal cord injury, to avoid the potential effects of unstable mental, physical and medical conditions; (2) all participants were healthy and free from acute infections apart from the SCI-related dysfunctions; and (3) CSCI subjects were able to manually self-drive their wheelchairs during the entire race. Participants with CSCI, but not those with SCI, suffered paralysis of some upper extremity muscles. Thus, the apparent mass of exercising muscles during the wheelchair half marathon was, in general, smaller in the CSCI than in the SCI athletes. In CSCI, the medullary cardiovascular center does not control peripheral sympathetic nerve activities due to transection of the neural network at the cervical level. None of the participants took any medications during the study period that would affect the cardiovascular and endocrine responses. The Human Research Committee of Wakayama Medical University School of Medicine approved the study protocol (#2136).

### 2.2. Study Protocol

Blood samples were collected from the antecubital vein in the morning before the warm-up period for the race, immediately after the completion of the race (distance; 21.0975 km) and 1 h after the completion of the race. Absolute and differential leukocyte counts, serum concentrations of IL-1ra and IL-6, and plasma concentrations of adrenaline and noradrenaline were measured in all participants, except for absolute and differential leukocyte counts in one participant with SCI. Accordingly, the data of the above two parameters reported in this study relate to five subjects each from the CSCI and SCI groups.

### 2.3. Leukocyte Count

One milliliter of the whole blood sample was transferred into tubes containing ethylenediaminetetraacetic acid (EDTA)-2K^+^ for absolute and relative leukocyte counts conducted using an automatic blood cell counter (MEK-6400, Nihon Kohden, Tokyo, Japan).

### 2.4. Measurement of Serum IL-1ra and IL-6 Levels

Six milliliters of blood were transferred into serum-separating medium tubes to measure the concentrations of IL-1ra and IL-6. After being left on the bench for least 30 min, the tubes were spun at room temperature at 3500 rpm for 15 min. Serum was stored at −80 °C until analysis. A high-sensitivity chemiluminescent enzyme immunoassay (CLEIA) kit (Fujirebio Co., Tokyo, Japan) was used for measurement of the IL-1ra concentration in serum (sensitivity: 0.2 pgmL^−1^). All measurements were performed in duplicates.

### 2.5. Measurements of Plasma Concentrations of Adrenaline, Noradrenaline, and Cortisol

Each three milliliters of blood sample were stored in a chilled vacutainer containing EDTA-2Na^+^. Plasma was stored at −80 °C until analysis. Adrenaline and noradrenaline were extracted from the plasma using alumina and measured using high-performance liquid chromatography, using a modification of the procedure described by Hunter et al. [32]. The plasma concentration of cortisol was measured using a competitive solid phase I radioimmunoassay technique (Dainabot Lab, Tokyo, Japan).

### 2.6. Statistical Analysis

All group data were expressed as the mean ± standard deviation of the mean (SD). A two-way repeated-measures ANOVA was used to evaluate differences between groups, within time points, and interactions of groups with time. We used Tukey’s test as a post hoc test when the results of analysis of variance tests were significant. Differences in variables between CSCI and SCI at baseline were determined by the Student’s *t*-test. A *p*-value less than 0.05 denoted the presence of a significant difference. All statistical analysis were performed using SPSS (version 24.0; IBM, Chicago, IL, USA).

## 3. Results

All participants were male subjects and no female SCI subjects participated in the race. All the study athletes completed the race. The mean race time achieved by the SCI athletes (61.6 ± 12.5 min) was significantly shorter than that of the CSCI (83.4 ± 16.3 min, *p* = 0.049) (Table 1).

**Figure 1:**
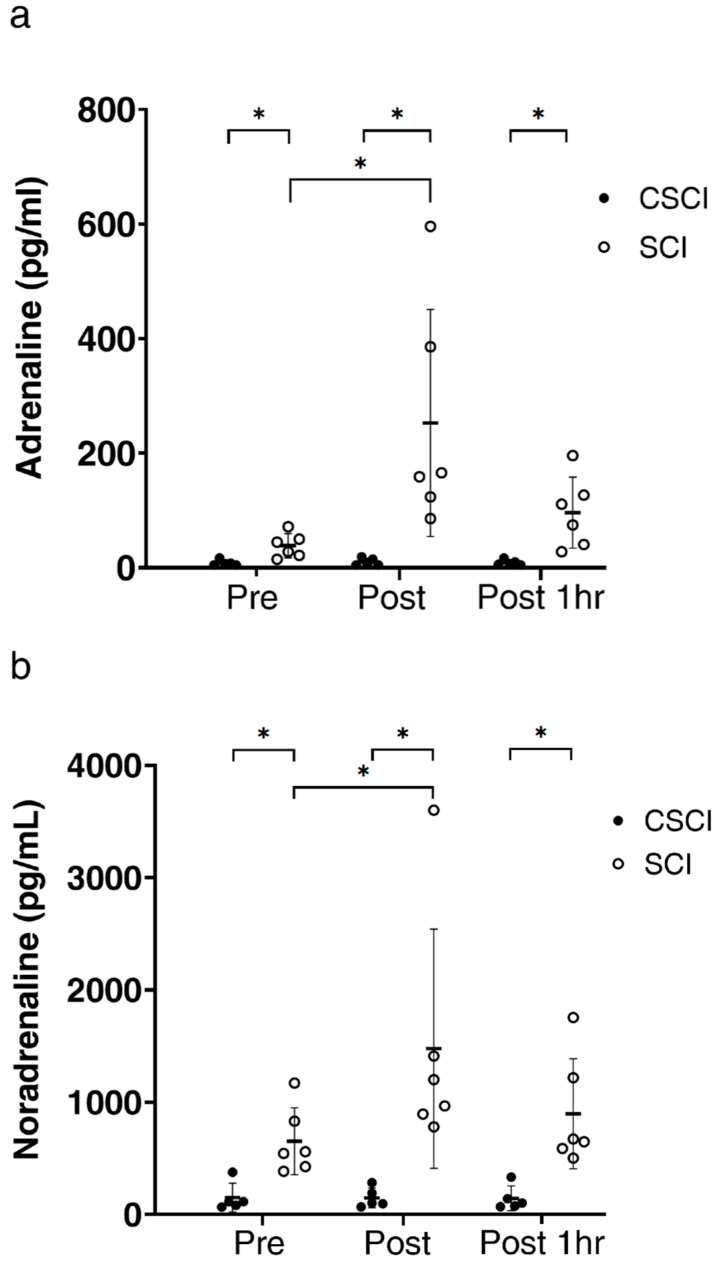
Plasma adrenaline (**a**) and noradrenaline (**b**) concentrations before (pre), immediately after (post) and one hour after wheelchair marathon race (post1hr) in athletes with thoracic and lumber spinal cord injury (SCI) and cervical spinal cord injury (CSCI). Thick horizontal lines with thin vertical lines: group mean ± SD values. * *p* < 0.05.

Plasma adrenaline and noradrenaline levels were significantly higher in SCI than CSCI throughout the study and both parameters increased significantly in SCI immediately after the race but recovered at 1 h after the race (Figure 1a,b).

**Figure 2:**
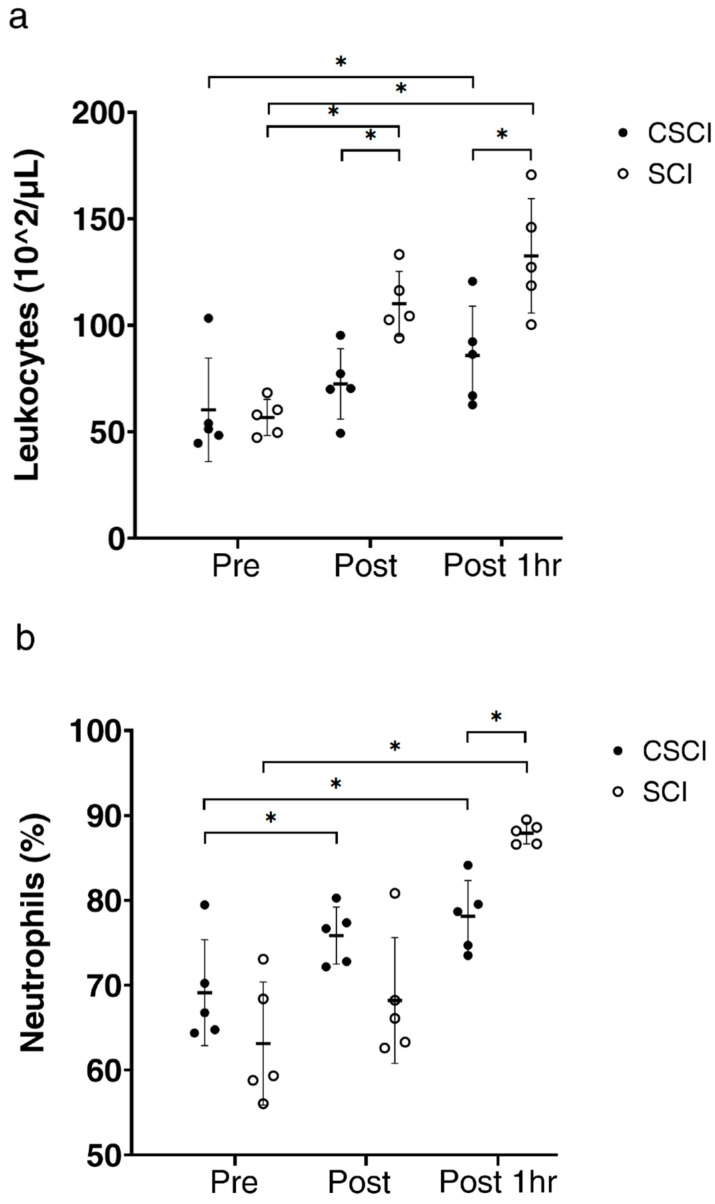
Leukocyte count (**a**) and percentage of neutrophils (**b**) before (pre), immediately after (post) and one hour after wheelchair marathon race (post1hr) in athletes with thoracic and lumber spinal cord injury (SCI) and cervical spinal cord injury (CSCI). Thick horizontal lines with thin vertical lines: group mean ± SD values. * *p* < 0.05.

There was no significant difference in the baseline leukocyte count between SCI (*n* = 5) and CSCI (*n* = 5) subjects (Figure 2a). The count increased significantly in SCI immediately after the race and 1 h after the race (*p* < 0.01, *p* < 0.05, respectively). The leukocyte count was significantly higher at 1 h after the race in CSCI, relative to the baseline (*p* < 0.01). Neutrophil counts were significantly higher immediately after the race and 1 h after the race in CSCI, relative to the baseline (*p* < 0.05, *p* < 0.01, respectively; Figure 2b). In SCI subjects, the neutrophil count was significantly higher at 1 h after the race, compared with the baseline (*p* < 0.01, Figure 2b).

**Figure 3:**
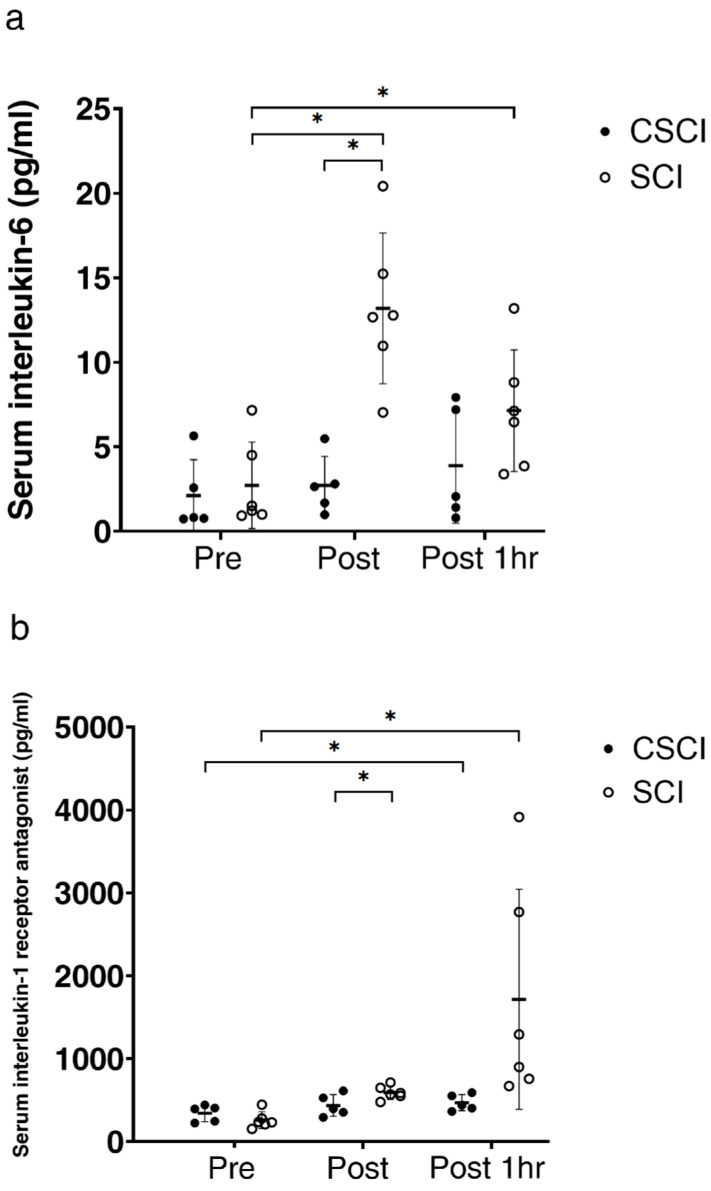
Levels of interleukin 6 (IL-6) (**a**) and interleukin-1 receptor antagonist (IL-1ra) (**b**) before (pre), immediately after (post) and one hour after wheelchair marathon race (post1hr) in athletes with thoracic and lumber spinal cord injury (SCI) and cervical spinal cord injury (CSCI). Thick horizontal lines with thin vertical lines: group mean ± SD values. * *p* < 0.05.

In SCI subjects, serum IL-6 concentrations were significantly higher immediately after the race (*p* < 0.01) and also at 1 h after the race (*p* < 0.05), compared with the baseline, whereas no significant changes in IL-6 were noted in CSCI (Figure 3a). Furthermore, the mean serum IL-6 level immediately after the race was significantly higher in the SCI than in the CSCI group (*p* < 0.01).

In both SCI and CSCI athletes, serum IL-1ra concentrations increased significantly at 1 h after the race, compared with the baseline (*p* < 0.05, Figure 3b). The mean serum IL-1ra level immediately after the race was significantly higher in SCI than in CSCI (*p* < 0.05, Figure 3b).

**Figure 4:**
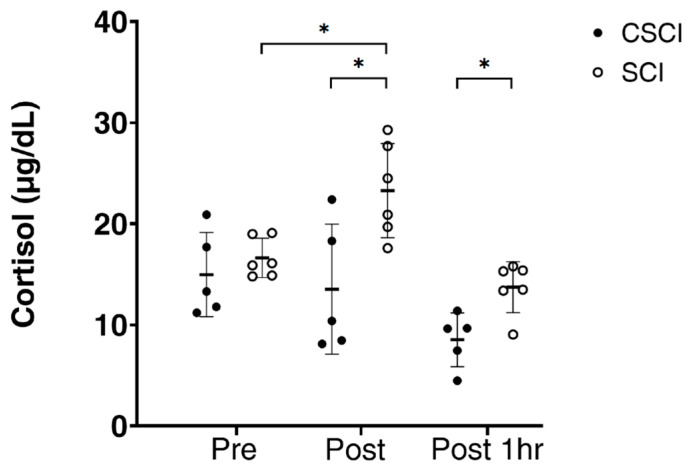
Cortisol levels measured before (pre), immediately after (post) and one hour after wheelchair marathon race (post1hr) in athletes with thoracic and lumber spinal cord injury (SCI) and cervical spinal cord injury (CSCI). Thick horizontal lines with thin vertical lines: group mean ± SD values. * *p* < 0.05.

In SCI subjects, serum cortisol concentrations were significantly higher immediately after the race (*p* < 0.01) compared with the baseline, whereas no significant changes in cortisol were noted in CSCI (Figure 4). Furthermore, the mean serum cortisol levels immediately after the race and 1 h after the race were significantly higher in the SCI than in the CSCI (*p* < 0.05, *p* < 0.01).

## 4. Discussion

The major findings of the present study were as follows: (1) IL-1ra immediately after the race was significantly higher in SCI than CSCI, and its level increased significantly at 1 h after the race in both groups; (2) the wheelchair marathon race neither increased adrenaline nor noradrenaline in CSCI; (3) serum IL-6 levels increased significantly in SCI but remained constant throughout the study in CSCI; the (4) neutrophil count at 1 h after the race was significantly higher in both groups, compared to the respective counts at baseline; and (5) the wheelchair marathon race increased cortisol levels in SCI but not in CSCI. These findings suggest that the wheelchair half marathon race increased IL-1ra even under stable IL-6 status in CSCI. The results also suggest that the increase in IL-1ra at 1 h after the race in CSCI is induced by peripheral blood neutrophils.

One of the most interesting results of the present study is the significantly higher levels of IL-1ra immediately after the race in SCI relative to those in CSCI and also the significant increase in IL-1ra at 1 h after the race in both SCI and CSCI. Ostowski et al. [10] measured IL-1ra, IL-6 and other cytokines in 10 male subjects who completed the 1997 Copenhagen Marathon before, immediately after the race and then every 30 min during the 4 h post-exercise recovery period. They found that the highest concentration of IL-6 occurred immediately after the race, whereas the IL-1ra level reached a peak value at 1 h post-exercise. Their findings suggested that cytokine inhibitors and anti-inflammatory cytokines limit the magnitude and duration of the inflammatory response to exercise [10] and that strenuous exercise induces an increase in IL-6, which is balanced by the release of the cytokine inhibitor IL-1ra.

To our knowledge, however, there have been no reports on the exercise-induced increase in IL-1ra in SCI and CSCI. In their study of SCI and CSCI wheelchair athletes, Paulson et al. [33] found no increase in IL-1ra in a submaximal exercise test followed by graded exercise to exhaustion on a motorized treadmill. Evidence suggests that the wheelchair half marathon is one of the most strenuous exercises for CSCI and SCI athletes and is associated with increases in IL-6 in CSCI and SCI [18,19].

IL-1ra was significantly higher in SCI than in CSCI immediately after the race. The intravenous infusion of recombinant human (rh)IL-6 in young healthy volunteers increased the rhIL-6 concentration to approximately 140 pg/mL, corresponding to the levels obtained during strenuous exercise, followed by the enhancement of IL-1ra levels [11]. These results and those of the 1997 Copenhagen Marathon [10] suggest that muscle-derived IL-6 plays a role in exercise-induced changes in IL-1ra. Therefore, significantly higher IL-1ra in SCI compared with CSCI is conceivable because the elevated IL-6 contributed to the increase in IL-1ra observed in SCI.

In the present study, circulating adrenaline and noradrenaline concentrations remained constant throughout the wheelchair half-marathon race in our subjects with CSCI. It is possible that this finding is related to the transection of the sympathetic nerves at the cervical level in these athletes, which connect the peripheral and central nervous systems. CSCI does not elicit sympathetic hyperactivity, and thus adrenaline and noradrenaline are not secreted by the adrenal medulla [2,34].

Pedersen and Febbraio [6] were the first to report that cytokines and other peptides are produced, expressed and released by muscle fibers (collectively termed myokines) during exercise. Umemoto et al. [1] studied IL-6 responses during hand ergometry exercise in SCI and reported significant increases in plasma IL-6 at 2 h after exercise of 60% VO2max. A wheelchair half marathon is known to result in a 9.4-fold increase in plasma IL-6 levels [18]. Our results on IL-6 in SCI are in agreement with those of previous studies [1,18]. Earlier studies demonstrated the upregulation of IL-6 mRNA in skeletal muscle biopsies and concluded that the contracting muscle fibers themselves were the source of IL-6 mRNA and protein, as confirmed by in situ hybridization and immunohistochemical analysis of biopsies from the human vastus lateralis muscles [35]. It seems that the magnitude of the exercise-related increase in plasma-IL-6 parallels exercise duration, exercise intensity, the muscle mass involved in the mechanical work, and endurance capacity [8]. Therefore, the mass of striated muscles in SCI is sufficient enough to increase IL-6 during the half marathon race.

With regard to CSCI, previous studies have reported significant increases in plasma IL-6 levels after a wheelchair half marathon [19]. However, the present results showed no increase in serum IL-6 during and after the wheelchair half marathon in CSCI. The amount of exercise-induced IL-6 secretion depends on the contracting muscle mass and activation by adrenaline [6,36,37]. The latter two parameters are lower in CSCI than SCI during exercise. The exercise-induced IL-6 secretion threshold in CSCI is delicately set under appropriate physiological and physical conditions. One methodological difference between the previous studies and our study is the ambient temperature on the day of the race. The average ambient temperature was 13.5 °C in the present study, which was lower than the 16.6 °C in the previous study [19]. Hashizaki et al. [38] reported that IL-6 increased when the core body temperature rose 1 °C in CSCI and concluded that ambient temperature was a significant determinant of an increase in IL-6. It is possible that the lack of increase in IL-6 observed in the present study was related to a lower average temperature relative to the critical temperature necessary to activate IL-6 secretion from muscle fibers.

In the present study, the percentage of neutrophils seemed to play an important role in the observed increase in serum IL-1ra at 1 h after the race in both groups of subjects. In able-bodied persons, neutrophil count is reported to increase during exercise and continue to increase after exercise [39,40]. We reported previously significant neutrophilia at 2 h after the race in CSCI and SCI athletes who completed a wheelchair half marathon [34]. What is the mechanism(s) of the observed increase in neutrophil count? The local response to tissue injury involves the production of cytokines that are released at the site of inflammation. These cytokines facilitate an influx of lymphocytes, neutrophils, monocytes and other cells, which participate in the process of tissue healing [10]. While we did not investigate these mechanisms in the present study, research-based evidence points to the potential roles of increase in local circulation, muscle damage, and heightened sympathetic nerve activity during exercise [41]. Further studies are needed to determine the exact mechanism(s) of exercise-induced neutrophilia in CSCI and SCI subjects.

The findings of the present study suggested the wheelchair half marathon race can increase IL-1ra even under a stable IL-6 status in CSCI subjects. Interestingly, IL-6 is known to stimulate the production of IL-1ra, which binds to and blocks the IL-1 receptor, thus exerting strong anti-inflammatory effects [42,43,44,45]. During exercise, the peak IL-1ra level occurs 1–2 h after the peak level of IL-6, suggesting that the level of IL-1ra reflects the production of IL-6 [10,42,43,44,45,46]. On the other hand, others have reported that various leukocyte phenotypes, including neutrophils, macrophages, fibroblasts, and monocytes, produce IL-1ra [21] and that IL-1ra protein is also produced by cells that can synthesize IL-1β, mainly hepatocytes, adipocytes, neutrophils and macrophages [5]. It is widely accepted that neutrophils constitutively produce IL-1ra [47]. Our findings suggest that the observed increase in IL-1ra in CSCI at 1 h after the race is most likely induced by peripheral blood neutrophils.

In our CSCI athletes, adrenaline, IL-6 and cortisol levels did not change throughout the race. Cortisol was significantly higher in SCI than in CSCI, both immediately and at 1 h after the race. The results suggest a lack of activation of the sympathetic nervous system and cortisol system in CSCI individuals during and after the wheelchair half-marathon race. In response to stress, the hypothalamus sends direct signals via the sympathetic nervous system to the adrenal glands, stimulating them to release various catecholamines, including adrenaline. Moreover, the hypothalamus secretes ACTH and this signals the cells in the adrenal cortex to produce and secrete corticosteroids [48]. An injection of IL-6 into humans increases plasma ACTH and plasma cortisol levels [11,30]. Moreover, both the pituitary corticotrophs and adrenocortical cells express IL-6 receptors, and IL-6 is reported to induce an increase in cortisol, both directly and indirectly [11,30]. One reason for the lack of elevated cortisol in CSCI may be that the constant level of IL-6 throughout the study.

One limitation of this study was that from a total of eight CSCI athletes who participated in the wheelchair half marathon, we were able to include only five from this group of athletes who were able to complete the race; the other three did not. Another study limitation was the study included only male athletes in this study; no female with SCI participated in the half marathon race. Thus, the results cannot be extrapolated to female athletes and further studies that include female SCI athletes are needed. Finally, our work does not categorically prove that the observed increase in serum IL-1ra in CSCI male athletes who completed the wheelchair half marathon is induced through circulating neutrophils. Further studies are needed to establish the roles of neutrophils and IL-1ra and confirm the present findings.

## 5. Conclusions

Both CSCI and SCI male athletes who completed the wheelchair half marathon race showed increased levels of IL-1ra, suggesting that this kind of strenuous exercise seems to increase IL-1ra in CSCI, even under a stable IL-6 status.

## Figures and Tables

**Table 1:** Anthropometric data of the study participants with spinal cord injuries.

	SCI Subjects	CSCI Subjects	*p* Value
*n*	6	5	-
Age (years)	44.2 ± 14.0	43.0 ± 10.4	0.89
BMI (kg/m^2^)	21.0 ± 2.4	22.2 ± 3.1	0.11
Spinal lesion	T3-L1	C5-8	-
Average race time (min)	61.6 ± 12.5	83.4 ± 16.3 *	<0.05

Data are mean ± SD values. * p < 0.05. SCI, spinal cord injury of thoracic and lumber regions; CSCI, cervical spinal cord injury.

## Data Availability

There is no data available to describe.

## References

[1] Umemoto Y., Furusawa K., Kouda K., Sasaki Y., Kanno N., Kojima D., Tajima F. (2011). Plasma IL-6 levels during arm exercise in persons with spinal cord injury. Spinal Cord.

[2] Nishimura Y., Nakamura T., Kamijo Y., Arakawa H., Umemoto Y., Kinoshita T., Sakurai Y., Tajima F. (2022). Increased serum levels of brain-derived neurotrophic factor following wheelchair half marathon race in individuals with spinal cord injury. J. Spinal Cord Med..

[3] McGuire D.K., Levine B.D., Williamson J.W., Snell P.G., Blomqvist C.G., Saltin B., Mitchell J.H. (2001). A 30-year follow-up of the Dallas Bedrest and Training Study: II. Effect of age on cardiovascular adaptation to exercise training. Circulation.

[4] Petersen A.M., Pedersen B.K. (2005). The anti-inflammatory effect of exercise. J. Appl. Physiol..

[5] Cauci S., Santolo M.D., Ryckman K.K., Williams S.M., Banfi G. (2010). Variable number of tandem repeat polymorphisms of the interleukin-1 receptor antagonist gene IL-1RN: A novel association with the athlete status. BMC Med. Genet..

[6] Pedersen B.K., Febbraio M.A. (2008). Muscle as an endocrine organ: Focus on muscle-derived interleukin-6. Physiol. Rev..

[7] Pedersen B.K., Steensberg A., Schjerling P. (2001). Muscle-derived interleukin-6: Possible biological effects. J. Physiol..

[8] Pedersen B.K., Akerstrom T.C., Nielsen A.R., Fischer C.P. (2007). Role of myokines in exercise and metabolism. J. Appl. Physiol..

[9] Pedersen B.K., Steensberg A., Fischer C., Keller C., Keller P., Plomgaard P., Febbraio M., Saltin B. (2003). Searching for the exercise factor-is IL-6 a candidate. J. Muscle Res. Cell Motil..

[10] Ostrowski K., Rohde T., Asp S., Schjerling P., Pedersen B.K. (1999). Pro- and anti-inflammatory cytokine balance in strenuous exercise in humans. J. Physiol..

[11] Steensberg A., Fischer C.P., Keller C., Møller K., Pedersen B.K. (2003). IL-6 enhances plasma IL-1ra, IL-10, and cortisol in humans. Am. J. Physiol. Endocrinol. Metab..

[12] Dinarello C.A. (1996). Biologic basis for interleukin-1 in disease. Blood.

[13] Dinarello C.A. (2011). Interleukin-1 in the pathogenesis and treatment of inflammatory diseases. Blood.

[14] Maedler K., Sergeev P., Ehses J.A., Mathe Z., Bosco D., Berney T., Dayer J.M., Reinecke M., Halban P.A., Donath M.Y. (2004). Leptin modulates β cell expression of IL-1 receptor antagonist and release of IL-1β in human islets. Proc. Natl. Acad. Sci. USA.

[15] Javan H., Li L., Schaaf C.L., Lee Y.S., Salama M.E., Dinarello C.A., Selzman C.H. (2022). Interleukin-1 Receptor Antagonism abrogates acute pressure overload-induced murine heart failure. Ann. Thorac. Surg..

[16] Hirose L., Nosaka K., Newton M., Laveder A., Kano M., Peake J., Suzuki K. (2004). Changes in inflammatory mediators following eccentric exercise of the elbow flexors. Exerc. Immunol. Rev..

[17] Nosaka K., Clarkson P.M. (1996). Changes in indicators of inflammation after eccentric exercise of the elbow flexors. Med. Sci. Sports Exerc..

[18] Sasaki Y., Furusawa K., Tajima F., Nakamura T., Kouda K., Kanno N., Kawasaki T., Umemoto Y., Shimizu K. (2014). Wheelchair marathon creates a systemic anti-inflammatory environment in persons with spinal cord injury. Clin. J. Sport Med..

[19] Ogawa T., Nakamura T., Banno B., Sasaki Y., Umemoto Y., Kouda K., Kawasaki T., Tajima F. (2014). Elevation of interleukin-6 and attenuation of tumor necrosis factor-α during wheelchair half marathon in athletes with cervical spinal cord injuries. Spinal Cord.

[20] Neiman D.C. (1997). Exercise immunology: Practical applications. Int. J. Sports Med..

[21] Colahan P.T., Kollias-Bakert C., Leutenegger C.M., Jones J.H. (2002). Does training affect mRNA transcription for cytokine production in circulating leucocytes?. Equine Vet. Suppl..

[22] Forti L.N., Van Roie E., Njemini R., Coudyzer W., Beyer I., Delecluse C., Bautmans I. (2017). Effects of resistance training at different loads on inflammatory markers in young adults. Eur. J. Appl. Physiol..

[23] Haller N., Reichel T., Zimmer P., Behringer M., Wahl P., Stöggl T., Krüger K., Simon P. (2023). Blood-based biomarkers for managing workload in athletes: Perspectives for research on emerging biomarkers. Sports Med..

[24] Cox A.J., Pyne D.B., Saunders P.U., Callister R., Gleeson M. (2007). Cytokine responses to treadmill running in healthy and illness-prone athletes. Med. Sci. Sports Exerc..

[25] Febbraio M.A., Pedersen B.K. (2002). Muscle-derived interleukin-6: Mechanisms for activation and possible biological roles. FASEB J..

[26] Pedersen B.K., Hoffman-Goetz L. (2000). Exercise and the immune system: Regulation, integration and adaptation. Physiol. Rev..

[27] Suzuki K., Nakaji S., Yamada M., Totsuka M., Sato K., Sugawara K. (2002). Systemic inflammatory response to exhaustive exercise. Cytokine kinetics. Exerc. Immunol. Rev..

[28] Park A., Anderson D., Battalion R.A., Nguyen N., Morse L.R. (2022). Ibuprofen use is associated with reduced C-reactive protein and interleukin-6 levels in chronic spinal cord injury. J. Spinal Cord Med..

[29] Allison D.J., Ditor D.S. (2015). Immune dysfunction and chronic inflammation following spinal cord injury. Spinal Cord.

[30] Bethin K.E., Vogt S.K., Muglia L.J. (2000). Interleukin-6 is an essential, corticotropin-releasing hormone-independent stimulator of the adrenal axis during immune system activation. Proc. Natl. Acad. Sci. USA.

[31] Hill E.E., Zack E., Battaglini C., Viru A., Hackney A.C. (2008). Exercise and circulating cortisol levels: The intensity threshold effect. J. Endocrinol. Invest..

[32] Hunter L.W., Rorie D.K., Yaksh T.L., Tyce G.M. (1998). Concurrent separation of catecholamines, dihydroxyphenylglycol, vasoactive intestinal peptide, and neuropeptide Y in superfusate and tissue extract. Anal. Biochem..

[33] Paulson T.A.W., Goosey-Tolfrey V.L., Lenton J.P., Leicht C.A., Bishop N.C. (2013). Spinal cord injury level and the circulating cytokine response to strenuous exercise. Med. Sci. Sports Exerc..

[34] Banno M., Nakamura T., Furusawa K., Ogawa T., Sasaki Y., Kouda K., Kawasaki T., Tajima F. (2012). Wheelchair half-marathon race increases natural killer cell activity in persons with cervical spinal cord injury. Spinal Cord.

[35] Hiscock N., Chan M.H., Bisucci T., Darby I.A., Febbraio M.A. (2004). Skeletal myocytes are a source of interleukin-6 mRNA expression and protein release during contraction: Evidence of fiber type specificity. FASEB J..

[36] Parrado A.C., Canellada A., Gentile T., Rey-Roldán E.B. (2012). Dopamine agonists upregulate IL-6 and IL-8 production in human keratinocytes. Neuroimmunomodulation.

[37] Batistaki C., Kostopanagiotou G., Myrianthefs P., Dimas C., Matsota P., Pandazi A., Baltopoulos G. (2008). Effect of exogenous catecholamines on tumor necrosis factor alpha, interleukin-6, interleukin-10 and beta-endorphin levels following severe trauma. Vasc. Pharmacol..

[38] Hashizaki T., Nishimura Y., Teramura K., Umemoto Y., Shibasaki M., Leicht C.A., Kouda K., Tajima F. (2018). Differences in serum IL-6 response after 1 °C rise in core body temperature in individuals with spinal cord injury and cervical spinal cord injury during local heat stress. Int. J. Hyperth..

[39] Pedersen B.K., Steensberg A. (2002). Exercise and hypoxia: Effects on leukocytes and interleukin-6-shared mechanisms?. Med. Sci. Sports Exerc..

[40] McCarthy D.A., Dale M.M. (1988). The leucocytosis of exercise. A review and model. Sports Med..

[41] Suzuki K., Yamada M., Kurakake S., Okamura N., Yamaya K., Liu Q., Kudoh S., Kowatari K., Nakaji S., Sugawara K. (2000). Circulating cytokines and hormones with immunosuppressive but neutrophil-priming potentials rise after endurance exercise in humans. Eur. J. Appl. Physiol..

[42] Ronsen O., Lea T., Bahr R., Pedersen B.K. (2002). Enhanced plasma IL-6 and IL-1ra responses to repeated vs. single bouts of prolonged cycling in elite athletes. J. Appl. Physiol..

[43] Dinarello C.A. (1994). Blocking interleukin-1 receptors. Int. J. Clin. Lab. Res..

[44] Horn F., Henze C., Heidrich K. (2000). Interleukin-6 signal transduction and lymphocyte function. Immunobiology.

[45] Tilg H., Trehu E., Atkins M.B., Dinarello C.A., Mier J.W. (1994). Interleukin-6 (IL-6) as an anti-inflammatory cytokine: Induction of circulating IL-1 receptor antagonist and soluble tumor necrosis factor receptor p55. Blood.

[46] Nehlsen-Cannarella S.L., Fagoaga O.R., Nieman D.C., Henson D.A., Butterworth D.E., Schmitt R.L., Bailey E.M., Warren B.J., Utter A., Davis J.M. (1997). Carbohydrate and the cytokine response to 2.5 h of running. J. Appl. Physiol..

[47] McColl S.R., Paquin R., Ménard C., Beaulieu A.D. (1992). Human neutrophils produce high levels of the interleukin 1 receptor antagonist in response to granulocyte/macrophage colony-stimulating factor and tumor necrosis factor alpha. J. Exp. Med..

[48] Lee D.Y., Kim E., Choi M.H. (2015). Technical and clinical aspects of cortisol as a biochemical marker of chronic stress. BMB Rep..

